# Study of Osteocyte Behavior by High-Resolution Intravital Imaging Following Photo-Induced Ischemia

**DOI:** 10.3390/molecules23112874

**Published:** 2018-11-04

**Authors:** Hengfeng Yuan, Wen Jiang, Yuanxin Chen, Betty Y. S. Kim

**Affiliations:** 1 Department of Neurosurgery, Mayo Clinic College of Medicine, 4500 San Pablo road, Jacksonville, FL 32224, USA; yuanfengfengggg@163.com (H.Y.); kim.betty@mayo.edu (B.Y.S.K.); 2 Shanghai Medical Schools, Fudan University, 138 Yixueyuan Road, Xuhui, Shanghai 200032, China; 3 Department of Radiation Oncology, UT Southwestern Medical Center, 6001 Forest Park Road, Dallas, TX 75235, USA; jiangwen0215@gmail.com

**Keywords:** osteocyte, osteonecrosis, two-photon, NADH

## Abstract

Ischemic injuries and local hypoxia can result in osteocytes dysfunction and play a key role in the pathogenesis of avascular osteonecrosis. Conventional imaging techniques including magnetic resonance imaging (MRI) and computed tomography (CT) can reveal structural and functional changes within bony anatomy; however, characterization of osteocyte behavioral dynamics in the setting of osteonecrosis at the single cell resolution is limited. Here, we demonstrate an optical approach to study real-time osteocyte functions in vivo. Using nicotinamide adenine dinucleotide (NADH) as a biomarker for metabolic dynamics in osteocytes, we showed that NADH level within osteocytes transiently increase significantly after local ischemia through non-invasive photo-induced thrombosis of afferent arterioles followed by a steady decline. Our study presents a non-invasive optical approach to study osteocyte behavior through the modulation of local environmental conditions. Thus it provides a powerful toolkit to study cellular processes involved in bone pathologies in vivo.

## 1. Introduction

Avascular osteonecrosis (AVN), commonly found in the regions of femoral head, is associated with bony cell death. Although AVN is attributed to ischemic insults, the exact molecular mechanism remains controversial [1,2]. Previous studies have suggested that intravascular thrombus and extravascular marrow lipid deposition can result in impaired blood supply leading to regional ischemia. The decreased oxygen tension ultimately causes osteocyte death and eventual collapse of subchondral bones [3,4]. Modern imaging techniques, such as magnetic resonance imaging (MRI), Single-photon emission computerized tomography (SPECT) and positron emission tomography (PET), have been utilized extensively in clinical settings to diagnose AVN [5,6,7,8]. However, imaging-based approaches that rely on optical platforms to study the cellular and molecular pathogenesis of AVN, possess unique technical challenges due to the high photon attenuation of calcified bone matrix.

Our current study aims to overcome many of the challenges associated with optical-based imaging of bony compartments and elucidates a potential molecular pathophysiology of AVN. Nicotinamide adenine dinucleotide (NADH), an important metabolic coenzyme in oxidative phosphorylation [9,10], is a sensitive non-linear proxy of tissue hypoxia [11,12]. Moreover, NADH is also a cofactor used by fatty acid synthetase in lipid biosynthesis, and by enzymes such as desmolases and hydroxylases in steroid biosynthesis [13]. NADH level within cells thus provides an accurate reflection of their metabolic demand and may serve as a surrogate marker for functional changes within osteocyte in AVN.

Due to the unique auto-fluorescence properties of NADH, imaging of NADH to monitor cellular metabolic states in vivo has been investigated in the past [14,15,16]. Recent studies have demonstrated that two-photon excitation microscopy may further enhance NADH imaging in vivo [16,17]. Using longer wavelength photons to excite NADH, less tissue scattering is expected, which will result in improved signal detection with reduced background noise. The small focal excitation volume further improves imaging resolution in the axial and longitudinal plane, thus enabling single cell imaging and tracking in deep tissue compartments such as the bone, marrow cavity or soft tissues [18,19,20,21].

Utilizing these advantages, our current study proposes a novel, all optical-based approach to generate and study the hypoxic microenvironment of AVN in situ. Performing high resolution real-time and long-term imaging of necrotic processes occurring in mouse calvaria, our goal is to characterize the deterministic relationships between vascular occlusion, osteocyte death, and changes in intracellular metabolic demand that contribute to the development of AVN.

## 2. Results

### 2.1. Real-Time In Vivo Imaging of Osteocyte Structure and Blood Vessels

**Figure 1:**
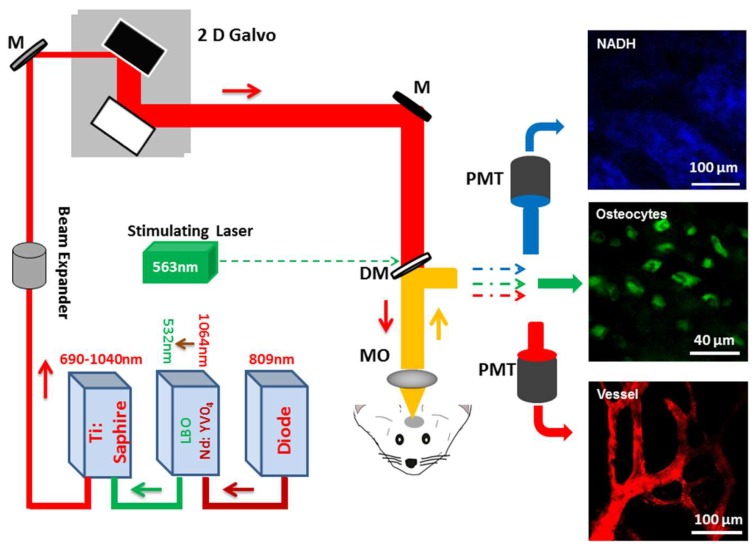
Schematic illustration of two photon imaging system. Abbrv: M, mirror; DC, dichroic mirror; MO, microscope objective; PMT, photomultiplier tube; 2D Galvo, two dimension galvanometer

**Figure 2:**
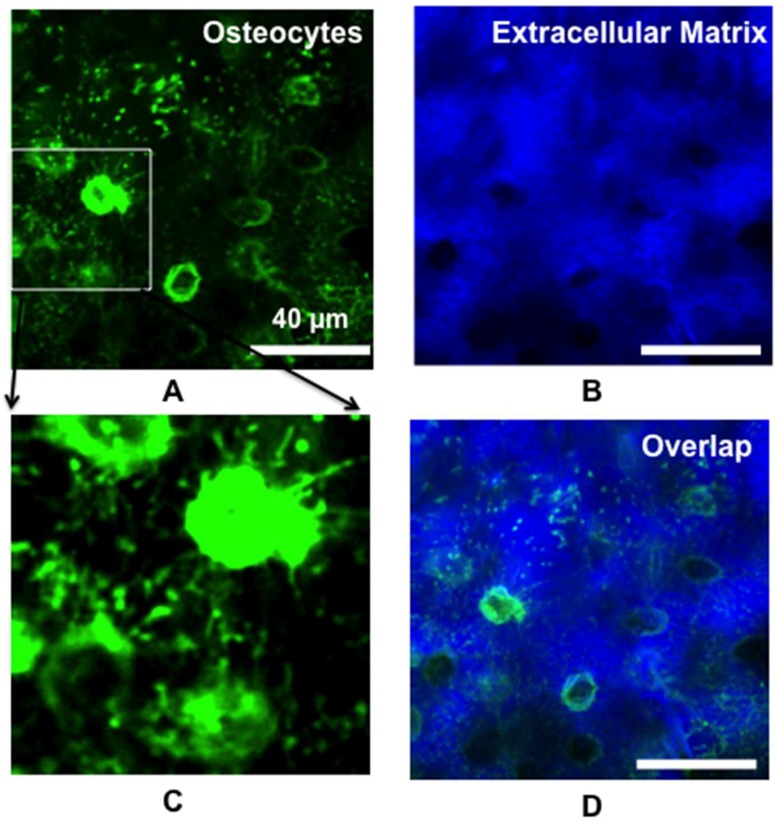
Intravital two-photon imaging of osteocytes: (**A**) Osteocytes were connected to one another through a network of cytoplasmic projections; (**B**) Extracellular matrix; (**C**) Magnification of the inset in Figure 2A; (**D**) Overlap of Figure 2A,B.

To track and visualize the live osteocytes and vasculature in the bone, a common dye, Calcein AM, was subcutaneously administrated (S.C, 20 mg/kg) 3 days before imaging and the dextran (70 kDa Texas red) intravenously injected 5 minutes before imaging (I.V, 10 mg/kg), Figure 1 showed the structure of the live osteocytes (green) and bone vasculature (red) using two-photon imaging system. We found that the live osteocytes were distributed and clustered along the vasculature (Supplementary Movie 1: Live osteocytes were distributed and clustered along the vasculature). In order to further confirm that the observed area was limited to the bone, we magnified the image area to visualize the osteocytes and extracellular matrix component within the bone (Figure 2). We observed that osteocytes were connected to one another through a network of cytoplasmic projections, including disk-shaped osteocytic lacunae and an abundance of dendritic canaliculus. Furthermore, the skull had been removed and the cortex exposed. The image from the cortex showed no labeled cells with Calcein dye (data not shown), which indicated that Calcien was specific to target and label osterocytes and imaging area did not exceed the range of bone.

### 2.2. Photo-thrombosis of the Selected Blood Vessels

**Figure 3:**
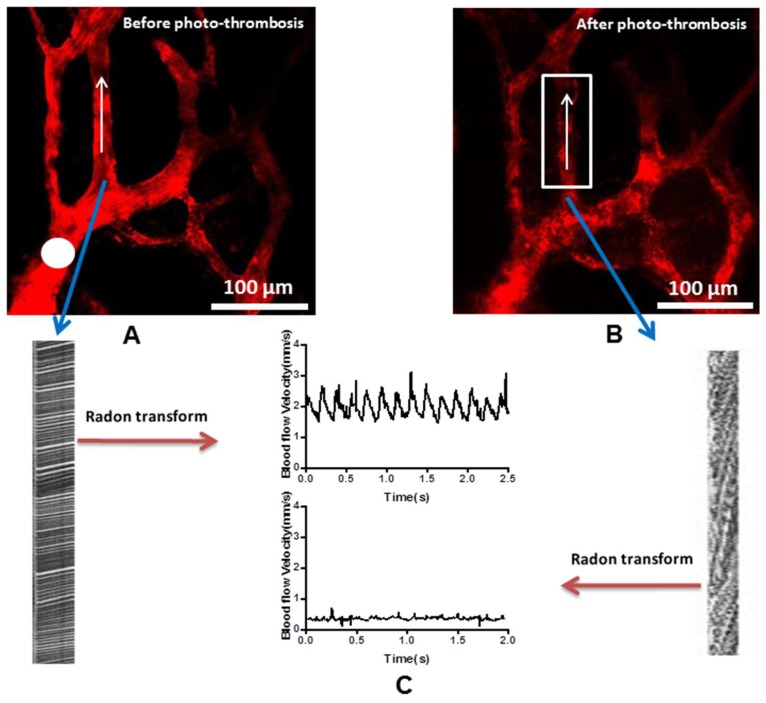
Photo-thrombosis of the arterioles: (**A**) Before photo-thrombosis, white circle demonstrates the target site of the laser beam; (**B**) after photo-thrombosis, many black dots (un-labeled RBC) were noted within the vessel; (**C**) velocity measurement of the blood flow.

To investigate the effects of blood flow supply on osteocytes metabolism, laser beam (563 nm) was used to target and stimulate the arterioles (white circle in Figure 3A) to make photo-thrombosis ischemic models after the intravenously administration of photosensitive dye, Rose Bengal (RB, I.V). After photo-stimulation, many black dots (un-labeled RBC) were found within the vessel (Figure 3B) with a reduction in blood flow and in some instances completely halted with the velocity under 0.2 mm/s (Figure 3C, Supplementary Movies 2 and 3: blood flow before and after photo-thrombosis, respectively).

### 2.3. In Vivo Quantitation of Osteocyte Metabolism

**Figure 4:**
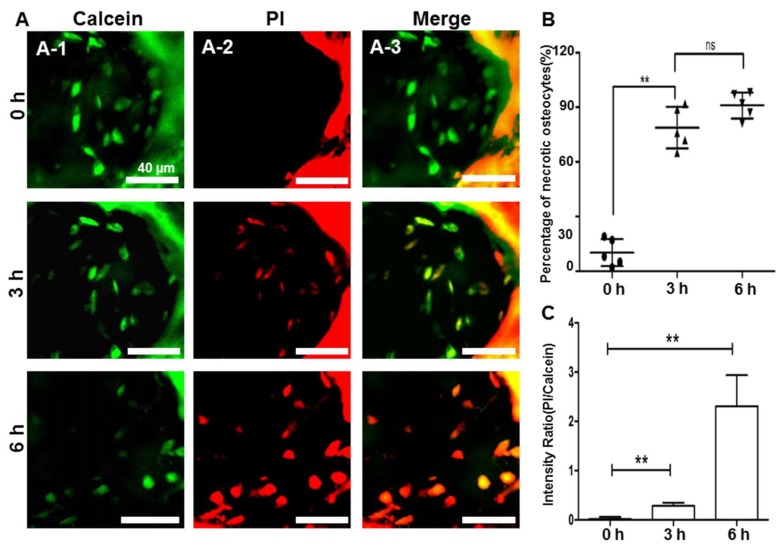
The changes of osteocytes after photo-thrombosis: (**A**) Osteocytes before they were stained with Calcein and PI at 0 h 3 h and 6 h after photo-thrombosis; (**B**) Percentage of dead osteocytes; (**C**) The intensity ratio of PI/Calcein. ** *p* < 0.01, by a two-tailed unpaired *t*-test; ns, not significant.

To study the changes of osteocytes metabolism under the ischemic and hypoxic microenvironment, propidium iodide (PI, sigma-aldrich, St. Louis, Missouri, USA; 1mg/ml) was intravenously administered to track the live cell state. We took images (shown in Figure 4A) at different time points (0, 3 and 6 h after photo-thrombosis). Our data indicated that before the photo-thrombosis, only a few stained cells with PI dye were captured (percentage <10%) (Figure 4B), which might be caused by the toxicity of staining dyes. Three hours after complete blood supply deprivation following photo-thrombosis, the percentage of PI stained dead cells were increased to approximately 75%, and fluorescence intensity ratio of PI/Calcein achieved 0.29 (Figure 4C). After 6 h, the dead cell rates reached nearly 95% and a PI/Calcein ratio of 2.31. In addition, the cell structure expanded due to cells lysis (Figure 4A, 6 h).

**Figure 5:**
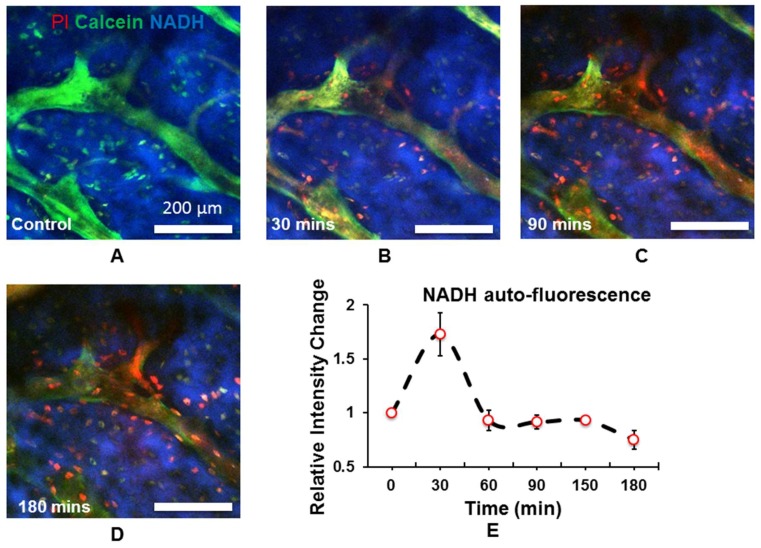
Intensity quantification of NADH auto-fluorescence after photo-thrombosis: (**A**–**D**) NADH auto-fluorescence (blue) changes inside the bone at the different time point (**E**) quantitative analysis of NADH auto-fluorescence change.

Simultaneously, NADH auto-fluorescence inside the bone had been collected under 740 nm laser beam excitation (Figure 5A–D). The NADH signal increased 30 min after the photo-thrombosis initial response to NADH accumulation caused by ischemia and hypoxia but decreased afterwards due to blood flow reperfusion (Figure 5E).

### 2.4. Hypoxic and Apoptotic Analysis of the Avascular Osteonecrosis Tissue

**Figure 6:**
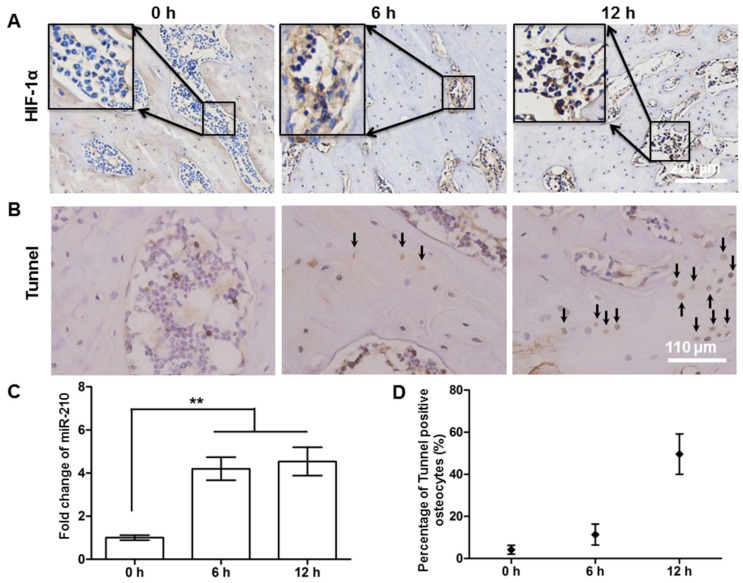
Histological examination of classic avascular osteonecrosis: (**A**) Immunohistochemical manifestation of HIF-1α in different postoperative stages; (**B**) tunnel staining of femoral heads, the positive osteocytes are shown with black arrows; (**C**) HIF-1α mRNA levels analyzed by real-time PCR; (**D**) the percentage of Tunnel positive osteocytes. ** *p* < 0.01, by a two tailed unpaired *t*-test.

To further verify these findings using two-photon technique, a classic AVN model was performed with transverse osteotomy. All rats survived the operation. The results showed that after osteotomy, the avascular femoral head was located in the hypoxic condition with a significant increase in HIF-1α (Figure 6 A,C), a sensitive indicator for hypoxia. However, we did not observe apoptotic osteocytes until 6 h after the surgery (Figure 6 B,D), later than our observation using the photo-thrombosis model.

## 3. Discussion and Conclusions

The pathogenesis of AVN is complex and involves multiple cellular processes [22]. Previous studies have proposed ways to artificially induce AVN in animal models including pulsed delivery of corticosteroids [23,24]. However, these methods may cause side effects like thinning bones and fractures, and it is also unclear whether these models represent similar pathophysiology as AVN observed in patients. The model system used in our study overcomes these concerns through the use of a non-invasive thrombo-occlusion procedure to mimic and simulate AVN. The precise use of high intensity lasers to induce photo-thrombosis of specific arterioles enables close monitoring and tracking local responses. Recently intravital bone imaging system has been introduced to study bone homeostasis [25] and NADH was regarded as a marker in metabolism hemostasis [14,16]. Frikha-Benayed et al. also used NADH to measure metabolic oxidative stress in situ from hypoxia and found that under hypoxia (postmortem), NADH levels could be regarded as a marker to reflect metabolism change. However, they created extreme hypoxia during postmortem, which could not mimic a real hypoxic environment in a live animal and these findings were limited to the dead animals [17]. We also show that there is abundant vesselsdistributed within the bone area. This studyuses the photo-thrombosis approach to simulate avascular osteonecrosis (AVN) environment in live animals, and for the first time, NADH, is used as a new metabolic marker to investigate osteocytes metabolism under the hypoxia environment in a high spatial resolution using two photon microscopy systems. Coupled with high-resolution intravital microscopy, our entire setup enabled real time tracking and monitoring of osteocyte fate and subcellular metabolic responses towards local stress signals from the ischemia within the bony environment.

Osteocytes are the terminally differentiated osteoblasts and account for over 90% of the entire cells in bone tissue [26]. The changes in osteocyte quantity and function in the setting of AVN have been investigated previously [27,28]. However, most studies thus far relied on the histological or ex vivo analyses of tissue or cells to extract phenotypic changes. In situ, direct observation of osteocyte response in the setting of ischemic stress is lacking. Our study showed that in real-time, osteocytes that reside within bones form an interconnected network through cytoplasmic projections [29,30], as well preferentially line the perivascular space. This observation provided strong and direct evidence that given the close proximity of osteocytes to blood vessels, avascular condition likely influences the biological activities of osteocytes.

Sato et al. reported that in a rat model with hip dislocation and osteotomy, the osteocytes death occurred at 12 h and AVN appeared after 96 h [31]. Using the similar surgical technique, we observed a few osteocytes death occurred at 6 h and with the majority of cell death at 12 h, which was consistent with the previous study. However, with the photo-thrombosis technique, we observed osteocytes beginning to die at 3 h. In this study, different from chronic formation of AVN, the high intensity stimulation in photo-thrombosis process damaged endothelial cells in the vessel rapidly. Clotting formation within the arteries was noted within seconds after initiation of laser excitation. In this context, PI rather than Tunnel assay, was used to assess for cell viability which aimed to manifest the early changes of osteocytes, which may have attributed to the early detection of osteocytes dying. In addition, we examined the calvarium and compared it to the femoral head. The differences in vascular supply as well as underlying biology between femur and calvarium bones may explain part of the differences of osteocyte responses.

NADH was an intrinsic auto-fluorescent indicator for energy metabolism [16]. In this study, ischemic and hypoxic condition inside the bone gave rise to the increase of NADH levels within 30 min, suggesting that the disorder of energy metabolism was involved in the early pathogenesis of AVN. Within 30 min of photo-thrombosis, the blockade of electron transport chains increased the accumulation of NADH in the cell and increased fluorescence intensity. However, because photo-thrombosis is transient and reversible [32,33,34], reperfusion relieved the hypoxic environment and facilitated the transfer from reduced NADH state to the oxidized NAD state with no auto-fluorescence [17].

In summary, our study demonstrated that osteocyte structure and function can be imaged in vivo with two-photon microscopy by applying a combination of endogenous and exogenous fluorophores. Changes in signal can be monitored in real time after the photo-thrombosis procedure in the bone. We have shown that this technique could simultaneously facilitate qualitative examination of osteocyte morphology with detailed quantitative analysis of biological changes in cell function, which could build a more complete understanding of AVN pathophysiology in the future.

## 4. Materials and Methods

### 4.1. Animal Preparation

Six old C57BL/6J mice (6–9-month-old, male) were anesthetized by the inhalation of isoflurane (4% for induction; 1.5 to 2% for surgery, and 1 to 1.5% for imaging) and immobilized in a custom-made stereotactic apparatus. After anesthesia, dexamethasone and buprenorphine were subcutaneously administered to reduce inflammation and pain. Body temperature was monitored by a rectal probe and maintained at 37.0 °C by a heating blanket (Homeothermic blanket systems, Harvard Apparatus, Holliston, MA, USA). Hair was removed on the skull to expose the frontoparietal skull section. Experiments were performed only if the physiological variables remained within normal limits. All experiments were performed under animal experimental guidelines approval of Mayo Clinic (A3915, 2015) and Fudan University (No. DF730, 2017).

### 4.2. Bone Cell and Vasculature Labeling

Calcein AM (Ebioscience, USA; 20 mg/kg, S.C.) was injected 3 days before examination to track the live osteocytes [25]; and five min before imaging. Dextran (dextran, Texas Red, 70 kDa, Thermo Fisher Scientific., Waltham, MA, USA; 12.5 mg/kg) was systemically administered to visualize the vasculature and blood flow in the bone.

To further characterize the damaged cells due to impaired blood supply, 100 μL of a solution of propidium iodide (PI, Sigma-Aldrich, USA; 1 mg/mL) was intravenously injected into the animal to label the dead cells.

### 4.3. Multi-Photon Imaging System and Photo-Thrombosis

The upright laser scanning microscope (BX61WI, Olympus, Tokyo, Japan) attached to a Ti:sapphire pulsed laser system (80 MHz repetition rate, <100 fs pulse width, Spectra Physics, Santa Clara, CA, USA) and software (Fluoview1000, Olympus, Japan) was used for two-photon fluorescence imaging. 20× water immersion (NA, 1.00; WD, 2 mm, Olympus), and 40× water-immersion objectives (NA 0.80, WD; 3.3 mm, Olympus) were selectively chosen for fluorescence imaging in vivo. 830-nm irradiation wavelength was used to excite calcein and dextran, and emission light was differentiated and detected with 525/50 and 615/50 filters, respectively. The average laser power for imaging was <30 mW.

Based on vessel diameter and blood flow direction, arterioles were discriminated and chosen as photo-thrombosis targets. System administration of Rose Bengal (RB, Sigma-Aldrich, USA; 100 µL, 10 mg/mL) was used to induce photo-thrombosis on specific arterioles under 563 nm laser beam illumination (intensity, 0.8–1.6 mW and stimulation duration, 80–100 s). All the procedure finished in 15 min after RB injection.

### 4.4. NADH and Extracellular Matrix Imaging

For NADH imaging, Images were taken at 12-bit depth at a resolution of 512 × 512 pixels with a pixel dwell time of 4  µs. NADH and Texas-Red-dextran were excited at 740 nm and emission wavelength was separated and detected using 460/50 and 615/50 filter. In addition, due to the accumulation of minerals (calcium and phosphorus) and collagens in the bone, second harmonic generation could be used to image extracellular matrix distributions in the bone under the excitation beam at 890 nm.

### 4.5. Ischaemic Osteonecrosis Model by Osteotomy Surgery

Fifteen male Sprague-Dawley rats were used to build up the ischaemic osteonecrosis model with transverse osteotomy. The operation was performed according to the methods used by Sato et al. [31]. The right femoral head of each rat was made surgically avascular while the contralateral side served as the control. The animals were sacrificed at different postoperative stages and the femoral heads were obtained for histological examination and hypoxic analysis (HIF-1α). The procedure for the preparation of samples can be found in our previous report [35].

### 4.6. Data Analysis

Images were processed using open source software Fiji (NIH, https://fiji.sc/) and an accustomed written program with MATLAB (Version 8.5.0 R2015a, Mathworks, Natick, MA, USA). Registration had been taken to perform the feature-based alignment of images at the different time point. After image registration, the number of dead osteocytes and fluorescence intensity of NADH were calculated or measured at five random regions of interest following background subtraction. In addition, radon transform algorithm had been used to measure blood flow velocity in the bone [36]. The data were presented as mean ± SD if possible, and unpaired two-tailed t-test was used with a *p* value of <0.05 was deemed as statistically significant.

## Supplementary Materials

The following are available online.
